# Does adherence to evidence-based practices during childbirth prevent perinatal mortality? A post-hoc analysis of 3,274 births in Uttar Pradesh, India

**DOI:** 10.1136/bmjgh-2019-002268

**Published:** 2020-09-14

**Authors:** Katherine EA Semrau, Kate A Miller, Stuart Lipsitz, Jennifer Fisher-Bowman, Ami Karlage, Bridget A Neville, Margaret Krasne, Jonathon Gass, Amanda Jurczak, Vinay Pratap Singh, Shambhavi Singh, Megan Marx Delaney, Lisa R Hirschhorn, Bhalachandra Kodkany, Vishwajeet Kumar, Atul A Gawande

**Affiliations:** 1 Ariadne Labs at Brigham and Women’s Hospital and Harvard TH Chan School of Public Health, Boston, Massachusetts, USA; 2 Division of Global Health Equity & Department of Medicine, Brigham and Women's Hospital, Boston, Massachusetts, USA; 3 Department of Medicine, Harvard Medical School, Boston, MA, United States; 4 Optum, Boston, Massachusetts, USA; 5 Department of Internal Medicine, Johns Hopkins University School of Medicine, Baltimore, Maryland, USA; 6 Department of Infectious Disease and Global Health, Cummings School of Veterinary Medicine, Tufts University, North Grafton, Massachusetts, USA; 7 Community Empowerment Lab, Lucknow, India; 8 Department of Medical Social Sciences, Northwestern University Feinberg School of Medicine, Chicago, Illinois, USA; 9 Institute for Global Health, Northwestern University Feinberg School of Medicine, Chicago, Ilinois, United States; 10 Jawaharlal Nehru Medical College, Belgaum, Karnataka, India; 11 Department of Surgery, Brigham and Women's Hospital, Boston, MA, United States; 12 Department of Health Policy and Management, Harvard TH Chan School of Public Health, Boston, MA, United States

**Keywords:** maternal health, obstetrics, public health

## Abstract

**Background:**

Evidence-based practices that reduce childbirth-related morbidity and mortality are core processes to quality of care. In the BetterBirth trial, a matched-pair, cluster-randomised controlled trial of a coaching-based implementation of the WHO Safe Childbirth Checklist (SCC) in Uttar Pradesh, India, we observed a significant increase in adherence to practices, but no reduction in perinatal mortality.

**Methods:**

Within the BetterBirth trial, we observed birth attendants in a subset of study sites providing care to labouring women to assess the adherence to individual and groups of practices. We observed care from admission to the facility until 1 hour post partum. We followed observed women/newborns for 7-day perinatal health outcomes. Using this observational data, we conducted a post-hoc, exploratory analysis to understand the relationship of birth attendants’ practice adherence to perinatal mortality.

**Findings:**

Across 30 primary health facilities, we observed 3274 deliveries and obtained 7-day health outcomes. Adherence to individual practices, containing supply preparation and direct provider care, varied widely (0·51 to 99·78%). We recorded 166 perinatal deaths (50·71 per 1000 births), including 56 (17·1 per 1000) stillbirths. Each additional practice performed was significantly associated with reduced odds of perinatal (OR: 0·82, 95% CI: 0·72, 0·93) and early neonatal mortality (OR: 0·78, 95% CI: 0·71, 0·85). Each additional practice as part of direct provider care was associated strongly with reduced odds of perinatal (OR: 0·73, 95% CI: 0·62, 0·86) and early neonatal mortality (OR: 0·67, 95% CI: 0·56, 0·80). No individual practice or single supply preparation was associated with perinatal mortality.

**Interpretation:**

Adherence to practices on the WHO SCC is associated with reduced mortality, indicating that adherence is a valid indicator of higher quality of care. However, the causal relationships between practices and outcomes are complex.

**Funding:**

Bill & Melinda Gates Foundation.

**Trial registration details:**

ClinicalTrials.gov: NCT02148952; Universal Trial Number: U1111-1131-5647.

**Key questions**

**What is already known?**

- The WHO Safe Childbirth Checklist (SCC) recommends 28 evidence-based practices to be done at all births, such as preparation of clean supplies and proper use of oxytocin.
- A recent, large, randomized trial of the SCC in India found no effect on perinatal mortality even though more practices were done in the intervention arm.
- Few studies have directly connected mortality with individual practices at birth, or bundles of practices, because of the demanding data requirements.

**What are the new findings?**

- We looked at a subsample of births from the trial where childbirth care was directly observed for 18 practices and found few associations between perinatal mortality and any specific, single practice performed by the birth attendants.
- Instead, the total number of practices performed was related to the perinatal mortality risk: where more practices were done, mortality was lower.
- This relationship was strongest for practices of direct provider care, such as initiating breastfeeding or using oxytocin properly, rather than for having supplies prepared at the bedside.

**What do the new findings imply?**

- The causal relationship between specific birth practices and perinatal mortality is complex.
- The minimum set of the most critical practices is yet to be identified.
- Performance of the SCC practices may reflect facility-level characteristics, health system-level factors, or even external sociodemographic context.

## Introduction

Childbirth-related mortality remains a significant problem worldwide, especially in low- and middle-income countries.^1^ In many settings and across socioeconomic groups, women have shifted from home-based to facility-based delivery in order to access skilled birth attendance and effective, high-quality care—long-recognised as critical in reducing mortality.^2^ However, this shift has not substantially improved health outcomes, suggesting a significant gap in the range and quality of care delivered in facilities.^3–5^

Quality of care in maternal and newborn health can be a broad and complex issue.^6–8^ Recently, the WHO synthesised many aspects of quality of care in this field into a multidimensional framework, encompassing eight domains health systems should pursue to reduce maternal and neonatal harm.^9^ The first of these domains emphasises the importance of providing evidence-based clinical care during childbirth.

Research has provided clear evidence for specific labor-and-delivery practices that positively impact perinatal outcomes.^10 11^ For example, appropriate neonatal-resuscitation methods result in significant reduction in early neonatal mortality; breastfeeding reduces diarrhoeal-related morbidity in newborns.^11 12^ However, the evidence of impact associated with more complete packages or bundles of practices is more limited.^13 14^

The WHO Safe Childbirth Checklist (SCC) is a tool to support birth attendants in performing 28 birth practices known to be effective in reducing mortality. As part of the SCC development process, a literature review was completed to identify key practices targeting the seven leading killers of mothers and newborns.^15–19^ (see online supplementary information in reference 15) Not all of the identified practices could be included in the SCC, which aimed for parsimony and feasibility. The 28 practices that were included address preparation of appropriate supplies at the bedside and direct provider care.^15^ The BetterBirth programme is a coaching-based implementation of the Checklist, tested in a large-scale, matched-pair, cluster-randomised controlled trial in Uttar Pradesh, India. The trial assessed the programme’s impact on the performance of the practices listed on the Checklist and on perinatal mortality, maternal morbidity and maternal mortality.^20 21^ The trial demonstrated that the BetterBirth programme effectively increased adherence to practices, but did not significantly reduce mortality and morbidity across the full sample of mothers and neonates.^22 23^

10.1136/bmjgh-2019-002268.supp1 Supplementary data

In order to better understand the relationship between the performance of evidence-based practices during childbirth and perinatal outcomes, we conducted a post-hoc, exploratory, subgroup analysis on all daytime births in study facilities in one region, in which we directly observed and documented the care that birth attendants provided to labouring women and newborns, as well as 7-day health outcomes. We observed care from the woman’s admission to the facility for childbirth services until 1 hour post partum. We calculated perinatal, early neonatal and stillbirth rates where practices were performed and not (individually, in bundles of practices and by overall number of practices administered). For the purposes of this study and acknowledging the results of the main trial, we pooled data from both intervention and control sites for analysis.

## Methods

### Study setting

We conducted the BetterBirth trial in primary and community health facilities in Uttar Pradesh, India. With the highest population of any Indian state (204 million, 77% rural) and a chronically elevated Neonatal Mortality Rate (32 per 1000 live births)^24^ and Maternal Mortality Ratio (258 per 100 000 live births),^25^ Uttar Pradesh is a high-priority region for improving the quality of childbirth care. The main trial included 120 facilities (60 in the intervention arm, receiving a coaching-based implementation of the Checklist, and 60 in the control arm, receiving the current standard of care) across 24 districts.^21 23^ All 120 facilities met predefined eligibility requirements; we matched facilities based on specific criteria (detailed elsewhere).^22^

### Data collection and outcomes

In a convenience sample of 30 facilities (15 intervention and 15 control) in the region around Lucknow (the state capital), trained nurses, independent of both the facilities and the intervention coaching staff, directly observed care delivered to mothers and newborns. On average, these 30 facilities had 2000 deliveries per year (5.5 per day) and 4.6 skilled birth attendants per facility, with only one to two attendants per 8-hour shift. In these health facilities, the majority of deliveries are attended by nurses and auxiliary nurse midwives; rarely, doctors provided delivery care.^26^ Intensive training of data collectors/observers, including an 8-day orientation period, training visits to the field, periodic refresher trainings and a comprehensive Data Quality Assurance Protocol was used to ensure high data quality.^27^ Data collectors were supervised; according to the data quality assurance protocol, each data collector had to achieve 100% accuracy and concordance with their supervisors on a subset of observations that were doubly observed (three deliveries every quarter per observer) and documented (20 forms every quarter per observer). Observers recorded data on all practices within a specific pause point (from admission, just before delivery, within 1 min of delivery and within 1 hour of delivery); deliveries were observed for one or more pause points. Observation of practices was limited to practical and observable interactions between provider and patient or provider actions to ready supplies.^20^ Some of the key practices measured were initial steps in a cascade of care. For example, taking of blood pressure and other vital sign assessment could be observed; however, technical quality of the vital sign assessment and follow-on clinical activities were not assessed. Independent observers also first recorded data on standardised paper forms and subsequently entered the data into an application on the same day after leaving the patient care area. Again, through a data quality assurance process, 20 forms per data collector were double-entered by the team leader; this was repeated until 100% concordance between data entry by data collector and their supervisor was achieved. Data collected by independent observers were not shared with facility staff.

Between 7 November 2014 and 23 November 2016, these independent observers documented birth attendants’ adherence to Checklist practices over 12-hour (daytime) shifts when intervention staff were not present. During their shifts, data collectors observed all or almost all women in labour and their birth attendants from admission until 1 hour post partum; they did not intervene in clinical care. We did have a notification system to raise awareness of concerns or in the setting of an emergency.

We followed women and their newborns that received care in the BetterBirth facilities and gathered data on 7-day health outcomes (perinatal mortality, maternal morbidity and maternal mortality). We used data from facility registers to document mortality and morbidity occurring at the facility. To assess 7-day health outcomes, we employed a call centre to reach women and their families by mobile phone between 8 and 42 days post partum. In cases in which the call centre could reach neither the woman nor a family member by 22 days post partum, a fieldworker conducted a home visit, resulting in ascertainment of outcomes.^28^

We assigned unique identifiers to all trial participants whose deliveries were observed; we used the same identifier to record health-outcomes data, allowing us to link the care provided to each mother/newborn pair with that pair’s outcomes.

### Data analysis

We calculated descriptive statistics of the facilities, the mothers and newborns and the levels of adherence to each practice documented by independent observers. In total, we observed 18 practices from admission until 1 hour post partum (table 1). Because five of these practices would not be performed in case of a stillbirth, we compared the ‘Full’ set of 18 practices to early neonatal mortality (death of a liveborn infant within 7 days post partum), which excludes stillbirths. A ‘Narrow’ set of 13 practices ought to be performed in all cases, thus we compared these practices to (i) perinatal mortality (death of an infant within 7 days post partum), which includes stillbirths, and (ii) stillbirths alone. We further subdivided the practices into two bundles: supply preparation, which encompasses both the physical availability of the supply and its preparation bedside, and direct provider care of patients, such as taking a temperature or administering oxytocin. We expected that the direct care practices would have a closer relationship to outcomes than supply preparation behaviours.

**Table 1:** Definitions of measured practices by set and bundle and outcome comparison

Patient population	‘Full’ set			‘Narrow’ set		
	All labouring women with a liveborn neonate			All labouring women		
Outcome assessed	Early neonatal mortality (stillbirth excluded)			(i) Perinatal mortality (stillbirth included) (ii) Stillbirth alone		
Practice	Included	Supply preparation	Direct care	Included	Supply preparation	Direct care
Partograph started	X		X	X		X
No oxytocin administered prior to delivery				X		X
Oxytocin administered to mother	X		X			
Maternal temperature taken	X		X	X		X
Maternal blood pressure taken	X		X	X		X
Newborn weight taken	X		X			
Newborn temperature taken	X		X			
Skin-to-skin initiated at birth	X		X			
Skin-to-skin maintained for 1 hour	X		X			
Initiation of breastfeeding within 1 hour	X		X			
Clean scissors/blade prepared	X	X		X	X	
Cord tie prepared	X	X		X	X	
Mucous extractor prepared	X	X		X	X	
Neonatal bag and mask prepared	X	X		X	X	
Pads prepared	X	X		X	X	
Clean towel prepared	X	X		X	X	
Proper hand hygiene*	X		X	X		X
Birth companion present at admission	X			X		
Birth companion present during pushing	X			X		
**Total number of practices**	**18**	**6**	**10**	**13**	**6**	**5**

*Proper hand hygiene defined as the birth attendant using gloves, soap and water OR gloves and alcohol rub.

Adherence to each individual Checklist practice was calculated as a per cent of births observed, with 95% CIs adjusted for clustering by facility. To examine the relationship between each individual practice and mortality, we calculated each mortality rate (deaths/1000 cases) among births where the practice was performed and those where it was not. We do not statistically test the differences between these rates due to the presence of many zero cells as well as to avoid multiple testing.

To graphically display the relationship between the total number of practices performed and mortality, we fit generalised additive models with cubic splines.^29^ These semi-parametric models make few assumptions about the underlying functional form, and are convenient for visual display because they closely mirror the raw data, which is valuable for visual interpretation. However, these models do not produce single, convenient statistical summaries of the relationships. For this purpose, we estimated ORs using logistic regression models that predicted death from the count of practices performed at a birth. All models were limited to women who were observed for all appropriate time points between admission and 1 hour post partum, which excluded all women referred out of the facility and all caesarean sections. These models estimate change to the odds of death with each additional practice performed, with 95% CIs adjusted for clustering by fitting an overdispersion parameter in the estimation procedure. Because we pooled the data from the intervention and the control arms, our logistic models tested whether the relationship between behaviours and mortality could have been changed by the intervention itself. We also conducted sensitivity analyses to assess the impact of (i) missing observation data and (ii) mortality outside the 7-day follow-up period defined by the trial.

### Ethical compliance

We obtained consent from each facility’s leadership for trial participation and data collection on eligible mothers from facility registers. Birth attendants and facility staff verbally agreed to participate prior to trial initiation. Additionally, we obtained verbal consent from mothers or their surrogates in order to follow-up with a phone call or home visit, and consent was reconfirmed by data collectors at the beginning of the follow-up call/home visit. Independent observers obtained written consent from women or their surrogates and verbal consent from birth attendants prior to observation.

## Results

The 30 facilities in the subsample included 8 primary health centres, 18 community health centres and 4 first referral units. Across these 30 facilities, independent observers collected data on 3274 individual labouring women. On average, labouring women were 25·8 years old and had 2·3 previous pregnancies. The facilities in which they laboured were staffed with an average of 4·6 birth attendants, and nurses attended 81·6% of births. Women generally delivered shortly after admission: median time from admission to delivery was less than 2 hours (table 2).

**Table 2:** Demographic characteristics of facilities (n=30), women (n=3274) and their newborns observed during labour and delivery

**Maternal Characteristics**	**Frequency (%)**
Maternal Age (years) (n=3247)	
16–18	3 (0.09)
19–24	1241 (38.2)
25–29	1368 (42.1)
30–34	528 (16.3)
35+	107 (3.3)
Missing	27
Gravida (n=2824)	
1	990 (35.1)
2-4	1650 (58.4)
5-7	179 (6.3)
8+	5 (0.2)
Missing	450
Caste (n=3003)	
Schedule Tribe or Caste	1086 (36.2)
Other Caste	1320 (43.9)
Muslim or Other	597 (19.9)
Missing	271
Number of offspring (n=3274)	
Singleton	3251 (99.3)
Twins	22 (0.7)
Triplets	1 (0.0)
Missing	0
Birth Attendant (n=3274)^+^	
Doctor	543 (16.6)
Nurse	2672 (81.6)
Auxiliary Nurse Midwife	662 (20.2)
Other	152 (4.6)
Missing	0
Minutes from Admission to Birth (n=2372)	
Mean (SE)	225.62 (13.5)
Median (SE)	119.61 (10.4)
Missing	2
**Newborn Characteristics**	**Frequency (%)**
Low Birth Weight* (n=3126)	
No	2050 (65.6)
Yes	1076 (34.4)
Missing	148
Preterm Birth** (n=2950)	
No	2316 (78.5)
Yes	634 (21.5)
Missing	324
**Facility Characteristics**	**Mean (SE)**
Annual Delivery Load (n=30)	2001·0 (71.0)
Distance to District Hospital in km (n=30)	33.2 (2.0)
Annual Household Income in USD (n=30)	586.8 (31.8)
Number of Women Observed (n=30)	109.1 (2.7)

+Numbers do not sum to 100% as it was possible to have more than one birth attendant.

*Low birth weight defined as 2500 g and less; data from facility register.

†Preterm birth defined as less than 37 weeks gestation; data from facility register.

US$, United States dollar.

Adherence by birth attendants to evidence-based practices varied greatly (table 3). Birth attendants performed certain practices almost universally—both in supply preparation (eg, cord tie by the bedside) and direct provider care (eg, ensuring birth companionship or weighing the baby). However, some practices were performed in only half of the observed cases, including clean towel preparation prior to delivery, administering oxytocin within 1 min after delivery, and initiating skin-to-skin warming after delivery. Birth attendants rarely performed other practices such as using the partograph, ensuring proper hand hygiene, maintaining skin-to-skin for 1 hour and taking the baby’s temperature (table 3).

**Table 3:** Birth attendant adherence to individual checklist practices, adjusted for facility-level clustering

Checklist practice observed	Adherence n (%)
On admission n=2731	
Birth companion present	2725 (99.78)
Maternal blood pressure taken	832 (30.47)
Maternal temperature taken	661 (24.20)
Partograph started	14 (0.51)
Just before pushing n=2866	
Proper hand hygiene*	428 (14.93)
Oxytocin not administered	1266 (44.17)
Clean towel prepared	1515 (52.86)
Scissors/blade prepared	2382 (83.11)
Cord tie prepared	2853 (99.55)
Mucus extractor prepared	2736 (95.46)
Neonatal bag prepared	2744 (95.74)
Pads prepared	2111 (73.66)
Within 1 min of delivery n=2865	
Oxytocin administered	1435 (50.09)
Birth companion present	2858 (99.76)
Within 1 hour of delivery n=2781	
Baby weight taken	2421 (87.06)
Baby temperature taken	499 (17.94)
Skin-to-skin warming initiated	1166 (41.93)
Skin-to-skin warming maintained for 1 hour	220 (7.91)
Breastfeeding initiated	911 (32.76)
Anytime n=3274	
Referred to another facility	
No	3209 (98.01)
Yes, before delivery	35 (1.07)
Yes, after delivery (mother)	1 (0.03)
Yes, baby	29 (0.89)
Maternal temperature taken	929 (28.38)
Maternal blood pressure taken	1195 (36.50)
Magnesium sulfate given	7 (0.21)

*Proper hand hygiene defined as the birth attendant using gloves, soap and water OR gloves and alcohol rub.

Of the 3274 observed deliveries, 166 perinatal deaths (50·7 per 1000 live births) occurred. Of those deaths, 56 (33·7%) were documented as stillbirths and 110 were early neonatal deaths (66·3%).

Table 4 shows the early neonatal and perinatal mortality rates where each practice was performed or not (a column for stillbirth rates is contained in online supplementary appendix 2). Several of these estimates are unstable because of small cell counts for deaths; of the 18 comparisons in the early neonatal mortality column, four cases have zero deaths and another four have counts of 10 or fewer. Of the 13 comparisons in the perinatal mortality column, three cases have zero deaths and another three have 10 or fewer. Some comparisons appear to show substantial differences: the early neonatal mortality rate was 20.7 among babies who were weighed and 61.5 among those not weighed. However, this is due to reverse causality because early neonatal deaths are generally not weighed. In our view, two comparisons suggest plausible relationships with mortality: non-administration of oxytocin prior to birth (perinatal mortality rate 37.1 where oxytocin was not given vs 54.4 where it was given) and breastfeeding initiation (early neonatal mortality rate 17.6 where it was initiated vs 30.0 where it was not).

**Table 4:** Early neonatal mortality and perinatal mortality by practice adherence

	Early neonatal mortality rate (excludes stillbirth) deaths per 1000 live births		Perinatal mortality rate (stillbirth + early neonatal mortality) deaths per 1000 births	
	Practice performed (deaths/cases)	Practice not performed (deaths/cases)	Practice performed (deaths/cases)	Practice not performed (deaths/cases)
Birth companion present	34.8 (93/2675)	0.0 (0/6)	52.5 (143/2725)	0.0 (0/6)
Partograph started	0.0 (0/14)	34.9 (93/2667)	0.0 (0/14)	52.6 (143/2717)
Proper hand hygiene*	26.2 (11/420)	30.9 (74/2397)	44.4 (19/428)	47.2 (115/2438)
Oxytocin not administered before birth	N/A	N/A	37.1 (47/1266)	54.4 (87/1600)
Clean towel prepared	30.2 (45/1492)	30.2 (40/1325)	44.9 (68/1515)	48.9 (66/1351)
Clean blade prepared	29.9 (70/2342)	31.6 (15/475)	46.2 (110/2382)	49.6 (24/484)
Cord tie prepared	30.3 (85/2805)	0.0 (0/12)	46.6 (133/2853)	76.9 (1/13)
Mucus extractor prepared	30.5 (82/2692)	24.0 (3/125)	46.1 (126/2736)	61.5 (8/130)
Neonatal bag prepared	30.0 (81/2701)	34.5 (4/116)	45.2 (124/2744)	82.0 (10/122)
Clean pads prepared	28.4 (59/2076)	35.1 (26/741)	44.5 (94/2111)	53.0 (40/755)
Oxytocin administered post partum	26.0 (37/1421)	34.4 (48/1395)	N/A	N/A
Birth companion present (post partum)	30.3 (85/2809)	0.0 (0/7)	46.9 (134/2858)	0.0 (0/7)
Baby weight taken	20.7 (50/2417)	61.5 (22/358)	N/A	N/A
Baby’s temperature taken	16.1 (8/498)	28.1 (64/2277)	N/A	N/A
Skin-to-skin warming initiated	24.1 (28/1164)	27.3 (44/1611)	N/A	N/A
Skin-to-skin warming maintained for 1 hour	36.5 (8/219)	25.0 (64/2556)	N/A	N/A
Breastfeeding initiated	17.6 (16/909)	30.0 (56/1866)	N/A	N/A
Mother's blood pressure taken (at any time)	27.2 (32/1177)	38.2 (78/2041)	41.8 (50/1195)	55.8 (116/2079)
Mother’s temperature taken (at any time)	29.4 (27/918)	36.1 (83/2300)	40.9 (38/929)	54.6 (128/2345)

N/A: Practices not appropriate to be conducted in case of stillbirth.

*Proper hand hygiene defined as the birth attendant using gloves, soap and water OR gloves and alcohol rub.

**Figure 1:**
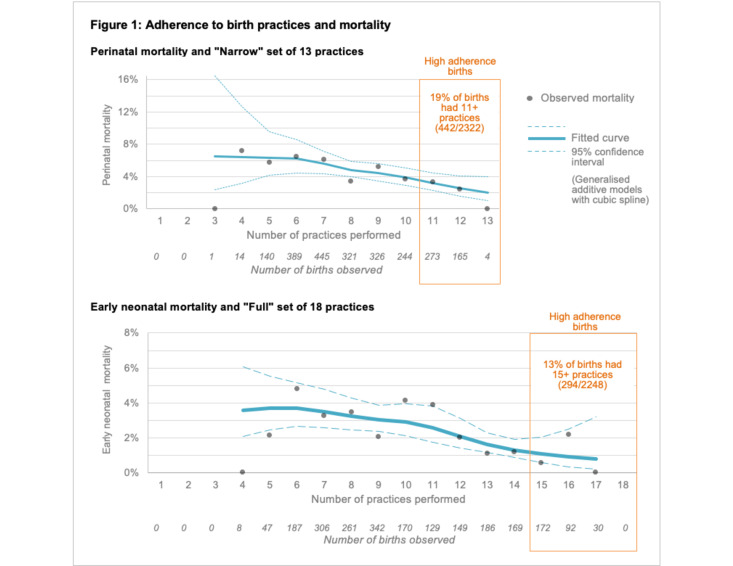
Cumulative adherence to practice and perinatal mortality.

Figure 1 shows perinatal and early neonatal mortality by the total count of behaviours performed at each birth, regardless of which specific behaviours were done. The total count of practices was never less than three because birth companions and prepared cord ties were nearly universal. The boxed areas indicate births with a high degree of adherence, or where 85% or more of the set of practices were done. For the ‘Narrow’ set, 19% of births had 11 or more behaviours, and for the ‘Full’ set, 13% of births had 15 or more behaviours. The fitted spline curves and 95% CIs clearly suggest an overall downward trend in mortality with more behaviours performed. To summarise this relationship parametrically, we fit the logistic models (table 5.) For each additional practice performed in the ‘Full’ set of practices for live births, we observed a 22% decrease (OR: 0·78, 95% CI: 0·71, 0·85) in the odds of early neonatal mortality by 7 days post partum. For each additional practice performed in the ‘Narrow’ set of practices for any birth, we observed an 18% decrease in the odds of perinatal mortality (OR: 0·82, 95% CI: 0·72, 0·93). We also saw a 19% decrease in the odds of stillbirth alone (table 5), which was not statistically significant (OR: 0·81, 95% CI: 0·64, 1·04). We found that interaction terms between study arm and count of practices were not significant, indicating no effect modification due to the intervention. Further sensitivity analyses did not show differences from the main analysis; thus, details are not reported here.

**Table 5:** Relationship between adherence to each additional practice and odds of mortality in 7 days post partum

				Bundle of practices					
	Any practice			Supply preparation			Direct provider care		
	No. of practices in set	OR (95% CI)	P value	No. of practices in set	OR (95% CI)	P value	No. of practices in set	OR (95% CI)	P value
‘Full’ set: practices for liveborn infants (early neonatal mortality)	18*	0.78 (0.71,0.85)	<0.01	6	0.95 (0.76, 1.19)	0.61	10	0.67 (0.56, 0.80)	<0.01
‘Narrow’ set: practices for any birth (perinatal mortality)	13*	0.82 (0.72, 0.93)	<0.01	6	0.88 (0.74, 1.05)	0.14	5	0.73 (0.62, 0.86)	<0.01
‘Narrow’ set: practices for any birth (stillbirth)	13*	0.81 (0.64, 1.04)	0.10	6	0.79 (0.56, 1.10)	0.15	5	0.83 (0.57, 1.21)	0.31

*Birth companions during admission and within 1 hour of delivery are included in the full set of practices, but not in the supplies or direct provider care practices.

Results by bundle of practices show that the direct provider care practices drove the overall association with early neonatal and perinatal mortality. The total number of supplies prepared was not associated with reduced perinatal mortality, early neonatal mortality or stillbirth (table 5). However, direct provider care demonstrated significant associations with reduced odds of mortality. For each additional direct provider care practice performed in the ‘Full’ set of practices for live births, we observed a 33% reduction (OR: 0·67; 95% CI 0·56, 0·80) in the odds of early neonatal mortality. For each additional direct provider care practice performed in the ‘Narrow’ set of practices for any birth, we observed a 27% reduction (OR: 0·73; 95% CI 0·62, 0·86) in the odds of perinatal mortality (table 5).

## Discussion

Our subanalysis of the BetterBirth trial in Uttar Pradesh, India—a resource-limited setting with high childbirth-related mortality rates—demonstrates that increased adherence to a bundle of evidence-based practices was associated with lower odds of early neonatal mortality and perinatal mortality. While the provision of any single, specific practice was not associated with a reduced odds of mortality, increasing the cumulative number of practices performed was significantly associated with a lower odds of early neonatal mortality by 22% and perinatal mortality (including stillbirth) by 18%, for each additional completed practice. Moreover, while the supplies identified by the WHO SCC are necessary to provide safe, effective childbirth care, supply availability alone was insufficient; increased supply preparation at the time of delivery was not associated with reduced odds of mortality. In contrast, increasing adherence to direct provider care practices was strongly associated with reduced odds of mortality. Rather than noting the impact of a specific practice, the analysis may be capturing an overall relationship: births where few practices are completed tend to have higher mortality than those where many practices are done.

The findings of this subanalysis offer insight into the primary outcome of the BetterBirth trial. In the trial, the BetterBirth programme—a coaching-based implementation of the WHO SCC—generated a notable difference in the performance of practices in intervention facilities compared with control facilities; however, we saw no difference in 7-day health outcomes for mothers or newborns.^22^ One potential conclusion from the trial is that the birth practices measured do not improve health outcomes for mothers and babies. We rejected this conclusion based on previous evidence and the face validity of practices like handwashing and appropriate use of oxytocin. Our findings in this subanalysis that the performance of more direct provider care behaviours associates strongly with improved perinatal outcomes supports that decision. Across the 60 intervention sites in the main trial, adherence to practices in the presence of the coach was similar in the 15 sites with independent observations compared with the 45 intervention sites where these observations were not done.^23^ However, the overall level of improvement in performance of evidence-based practices may still have been insufficient to achieve mortality impact. For example, in the main trial, practice adherence in the 15 intervention arm sites with observation reached an average of 72%. Further, the highest levels of adherence focussed on supply preparation at the time of delivery rather than direct provider care.^22 30^ While improving supply preparation at the bedside was an important outcome, this change alone would not generate a decrease in mortality without the provider-based behaviours. Second, it is possible that the practices observed and measured were not the most proximate practices required to prevent perinatal mortality or those due to the specific causes of death in this setting. Third, the relationship between practice adherence and outcomes may follow a threshold effect. In this subanalysis, less than one-fifth of the observed deliveries received >85% of observed practices, where the lowest mortality rates occurred. This threshold effect remains an area for further investigation, especially as other programmes implementing the SCC in Namibia and in Rajasthan and Karnataka, India, which have achieved >85% practice adherence have reported substantial reductions in stillbirth and in-facility mortality rates.^22 31 32^

More broadly, practice adherence is only one piece (although a crucial one) of safe, effective intrapartum care. Factors within the facility—unmeasured in the BetterBirth trial and in this subanalysis—undoubtedly impact patient outcomes; for example, technical quality of practices, practices unmeasured in this study, provider motivation, accessibility to caesarean section, referral systems and providers use of them to ensure timely access to higher level care, and communication between providers and mothers may impact their adherence to practices. Outside the facility, context plays a powerful role: the particular mix and intensity of health conditions that the population faces which impact the health of the pregnant woman, the availability and use of antenatal and postnatal care in the community, the timeliness of a woman’s arrival to the facility and the government economic and health-related policies, for example, conditional cash transfer for facility-based delivery. In a recent analysis from the BetterBirth trial, traditionally measured facility-level characteristics were not directly associated with mortality, however, general risk factors, such as women’s literacy and geographical location, were associated with mortality.^33^ All of these factors and many others certainly complicated the link between practice adherence and health outcomes in the BetterBirth trial.

Our findings suggest that focussing on vital changes in frontline clinical practice—specifically raising adherence to these evidence-based practices among birth attendants—offers an actionable approach: limited in scope but potentially impactful, measurable and achievable. Recognising that the SCC may omit certain evidence-based practices that are closely related to mortality in this setting, we call for further research to identify and test those practices. However, regardless of the specific practices involved, this research suggests that improving adherence to a bundle of practices—rather than one or two targeted practices—may be more likely to succeed in improving health outcomes. As with quality-improvement work in other areas of medicine, implementation may be challenging as convincing providers to shift their practices requires time, resources and persistence.^31 34–37^ Knowing that we must convince providers to change many behaviours rather than just a few does not make for an easier journey toward improved quality of care and decreased adverse health outcomes. Finally, this focus on adherence to practices must include strategies to establish and maintain an appropriately resourced, enabling environment to improve the technical and experiential quality of care and reduce mortality.^38 39^

In this subanalysis, some key limitations exist. First, independent observers recorded only completion (or non-completion) of the practice and not the technical quality performed. The practices listed on the WHO SCC and the observed practices presented here are not a comprehensive list of all care required for newborn survival, and a different package of practices may have led to a different result. As previously described, observation of this subset of practices offers a partial view in the care provided and Hawthorne effect may exist with an external observer.^40 41^ Second, we cannot assess causation in this study, only association between adherence to practices and health outcomes. Third, while we have attempted to address the potential for reverse causality in our analysis, we may not have completely controlled for it. As we do not have exact timing of death, reverse causality may create false associations in the case of a neonatal death shortly after birth; for example, taking of baby weight. We addressed this by examining three separate outcomes: stillbirth, early neonatal deaths and perinatal mortality. Fourth, we may have lacked power to detect impact of an individual practice due to relatively small numbers of deaths. Finally, we recognise the strong possibility that the performance of more practices was a proxy measure for contextual factors that were actually the root cause of both improved quality of care and improved outcomes. It is possible that practice adherence was an indicator of an underlying stronger health system at the facility level. In order to better design and implement interventions intended to decrease adverse health outcomes, we require further research to better understand whether this combination of practices is, in fact, only a proxy or also a contributing factor to improved outcomes. Using that information, we need to then identify and implement site specific interventions targeting the identified underlying causal factor(s) to reduce neonatal and maternal harm.

The connection between adherence to a bundle of evidence-based practices and mortality observed here supports the use of measuring adherence to evidence-based interventions in efforts to improve quality and effectiveness of care. However, our findings demonstrate that adherence is a necessary but insufficient component of the causal pathway to reduce perinatal mortality rates. In such a complex system, we found no single practice serving as a ‘magic bullet’ to reduce childbirth-related mortality. Our work supports broader efforts to address systems and other factors which may limit the impact of process-focussed interventions.
