# Supplementary material for: Does adherence to evidence-based practices during childbirth prevent perinatal mortality? A post-hoc analysis of 3,274 births in Uttar Pradesh, India
Source: BMJ Glob Health. 2020 Sep 14;5(9):e002268. doi: 10.1136/bmjgh-2019-002268 (PMC7490951; doi:10.1136/bmjgh-2019-002268)
Supplement: Supplementary data [file bmjgh-2019-002268supp001.pdf]

Supplementary Information for Manuscript: "Do evidence-based practices during childbirth prevent perinatal mortality? A post-hoc analysis of 3,3274 births in Uttar Pradesh, India"

Table A: Observed Mortality by Seven Days Postpartum

**Mortality N=3274**	**N(%)**
Perinatal mortality	166 (5·07)
Stillbirth	56 (1·71)
Early Neonatal Death	110 (3·36)

Table B: Stillbirth Rate (stillbirths per 1000 births) by Practice Adherence in 30 facilities in Uttar Pradesh, India

	Stillbirth Rate: Deaths per 1000 births	
	Practice Performed (deaths/cases)	Practice Not Performed (deaths/cases)
**Birth companion present**	18·5 (50/2702)	0·0 (0/6)
**Partograph Started**	0·0 (0/14)	18·6 (50/2694)
**Proper Hand Hygiene****	18·8 (8/426)	16·9 (41/2425)
**Oxytocin Not Administered Before Birth**	12·7 (16/1262)	20·8 (33/1589)
**Clean Towel Available**	15·3 (23/1508)	19·4 (26/1343)
**Clean Blade Available**	16·9 (40/2371)	18·8 (9/480)
**Cord Tie Available**	16·9 (48/2838)	76·9 (1/13)
**Mucus Extractor Available**	16·2 (44/2721)	38·5 (5/130)
**Neonatal Bag Available**	15·8 (43/2731)	50·0 (6/120)
**Clean Pads Available**	16·7 (35/2099)	18·6 (14/752)
**Oxytocin Administered Postpartum**	N/A	N/A
**Birth Companion Present (Postpartum)**	17·2 (49/2843)	0·0 (0/7)
**Baby Weight Taken**	N/A	N/A
**Baby's Temperature Taken**	N/A	N/A
**Skin-to-Skin Warming Initiated**	N/A	N/A
**Skin-to-Skin Warming Maintained for One Hour**	N/A	N/A
**Breastfeeding Initiated**	N/A	N/A
**Mother's Blood Pressure Taken (At Any Time)**	15·2 (18/1188)	18·5 (38/2060)
**Mother's Temperature Taken (At Any Time)**	11·9 (11/923)	19·4 (45/2325)
